# Chaski, a novel *Drosophila* lactate/pyruvate transporter required in glia cells for survival under nutritional stress

**DOI:** 10.1038/s41598-018-19595-5

**Published:** 2018-01-19

**Authors:** María Graciela Delgado, Carlos Oliva, Estefanía López, Andrés Ibacache, Alex Galaz, Ricardo Delgado, L. Felipe Barros, Jimena Sierralta

**Affiliations:** 1 Department of Neuroscience, Faculty of Medicine, Universidad de Chile, Santiago, Chile; 2 Biomedical Neuroscience Institute, Faculty of Medicine, Universidad de Chile, Santiago, Chile; 3 Department of Cell and Molecular Biology, Faculty of Biological Sciences, Pontificia Universidad Católica de Chile, Santiago, Chile; 4 Drosophila Ring in Developmental Adaptations to Nutritional Stress (DRIDANS), Universidad de Chile, Santiago, Chile; 5 Department of Biology, Faculty of Sciences, Universidad de Chile, Santiago, Chile; 6 Centro de Estudios Científicos, Valdivia, Chile

## Abstract

The intercellular transport of lactate is crucial for the astrocyte-to-neuron lactate shuttle (ANLS), a model of brain energetics according to which neurons are fueled by astrocytic lactate. In this study we show that the *Drosophila chaski gene* encodes a monocarboxylate transporter protein (MCT/SLC16A) which functions as a lactate/pyruvate transporter, as demonstrated by heterologous expression in mammalian cell culture using a genetically encoded FRET nanosensor. *chaski* expression is prominent in the *Drosophila* central nervous system and it is particularly enriched in glia over neurons. *chaski* mutants exhibit defects in a high energy demanding process such as synaptic transmission, as well as in locomotion and survival under nutritional stress. Remarkably, locomotion and survival under nutritional stress defects are restored by *chaski* expression in glia cells. Our findings are consistent with a major role for intercellular lactate shuttling in the brain metabolism of *Drosophila*.

## Introduction

The function of the nervous system requires a large supply of energy, as exemplified by the fact that in the mammalian brain, 50–60% of total ATP produced in the brain is used to support ion transport^1^; in flies, retinal photoreceptor cells consume about 10% of the ATP production of the whole animal^2^. Our current view about the field of brain energetics has evolved from a one centered in neurons into a one in which astrocytes and neurons play complementary roles to support the high demand of excitability and synaptic activity. In this view, the metabolic communication between neurons and glia is crucial to sustain brain function, relevating the need to unravel the mechanisms that underly this communication.

Within cells, several glucose-derived metabolic intermediates can subsequently be oxidized for energy production (i.e. lactate, pyruvate, glutamate, or acetate)^3^, while ketone bodies are mainly used during development and starvation^4,5^. In mammals, it has been estimated that over 10% of glucose entering the brain is converted to lactate despite normal oxygen levels, a metabolic process known as aerobic glycolysis^6^. Lactate production through aerobic glycolysis is a metabolic feature of astrocytes^7,8^. The high-energy demand as the result of glutamatergic synaptic activity is thought to stimulate aerobic glycolysis in astrocytes^9^ producing lactate that is secreted and used by neurons as energy source. This metabolic interaction has been termed the astrocyte-neuron lactate shuttle (ANLS) hypothesis^9,10^. Recent evidence from invertebrates supports that metabolic compartmentalization and coupling of neurons and glial cells is a conserved, fundamental feature of bilaterian nervous systems independent of their size^11^. Moreover, the lack of an apparent detrimental effect of glycolytic enzyme deletion in *Drosophila* neurons suggests that insects may have evolved an extreme version of ANLS, in which neurons would be fueled by lactate and/or alanine produced by glial cells^11^.

In vertebrates, lactate is co-transported with protons across the plasma membrane by members of the SLC16A family of monocarboxylic acid transporters (MCT)^12^, following the chemical gradient. The SLC16A family comprises 14 members; while four of them, MCT1 to 4 (encoded by SLC16A 1, 7, 8 and 3 respectively) have been characterized as lactate/pyruvate or ketone acids transporters^12^, others function as transporters for thyroid hormones and aromatic aminoacids; nevertheless, for most of them their substrates have not been identified yet nor their function as transporters has been confirmed^12^.

The *Drosophila* genome contains 15 genes with variable homology to members of the mammalian SLC16A family; several of them are expressed in the adult brain and none of them has been fully characterized (flybase.org).

Here, we describe the function of the gene CG3409 predicted to be an MCT, which we named *chaski* (*chk*) in honor to the Inca messenger (chasqui or *chaski*) that carried information to the different villages of the Inca Empire. Our results indicate that *chk* encodes a lactate/pyruvate transporter, which is expressed in larval and adult brain, and enriched in glial cells. We also show that, *in vivo*, global loss of *chk* function associates to defects in synaptic transmission and locomotion, as well to impaired survival during starvation. Most significantly, the reintroduction of *chk* only in glia cells restores the resistance to starvation and the locomotion activity. In summary, we describe here for the first time the function of an MCT expressed in *Drosophila* brain, highlighting the remarkable conservation of metabolic mechanisms in the brain and further supporting a role for lactate shuttling in the nervous system.

## Results

### *chaski* has the signature of a monocarboxylate transporter

*chk* (Flybase ID FBgn0033095, annotated as CG3409) was identified by homology to mammalian MCTs and selected among genes expressed in the adult fly brain (http://flybase.org/reports/FBgn0033095.html). The *chk* locus comprises three transcriptional units originated from three different transcription initiation starts, each containing two open reading frame (ORF). *chk* is the longest ORF with a 5093 nucleotides sequence encoding an 894 aminoacid protein with homology to the solute carrier family SLC16A, which includes lactate and pyruvate transporters in human; the second ORF encodes a predicted 165 aminoacid peptide (CG45092) with no homology to known proteins outside the *Drosophila* genus (see also Fig. S1).

**Figure 1:**
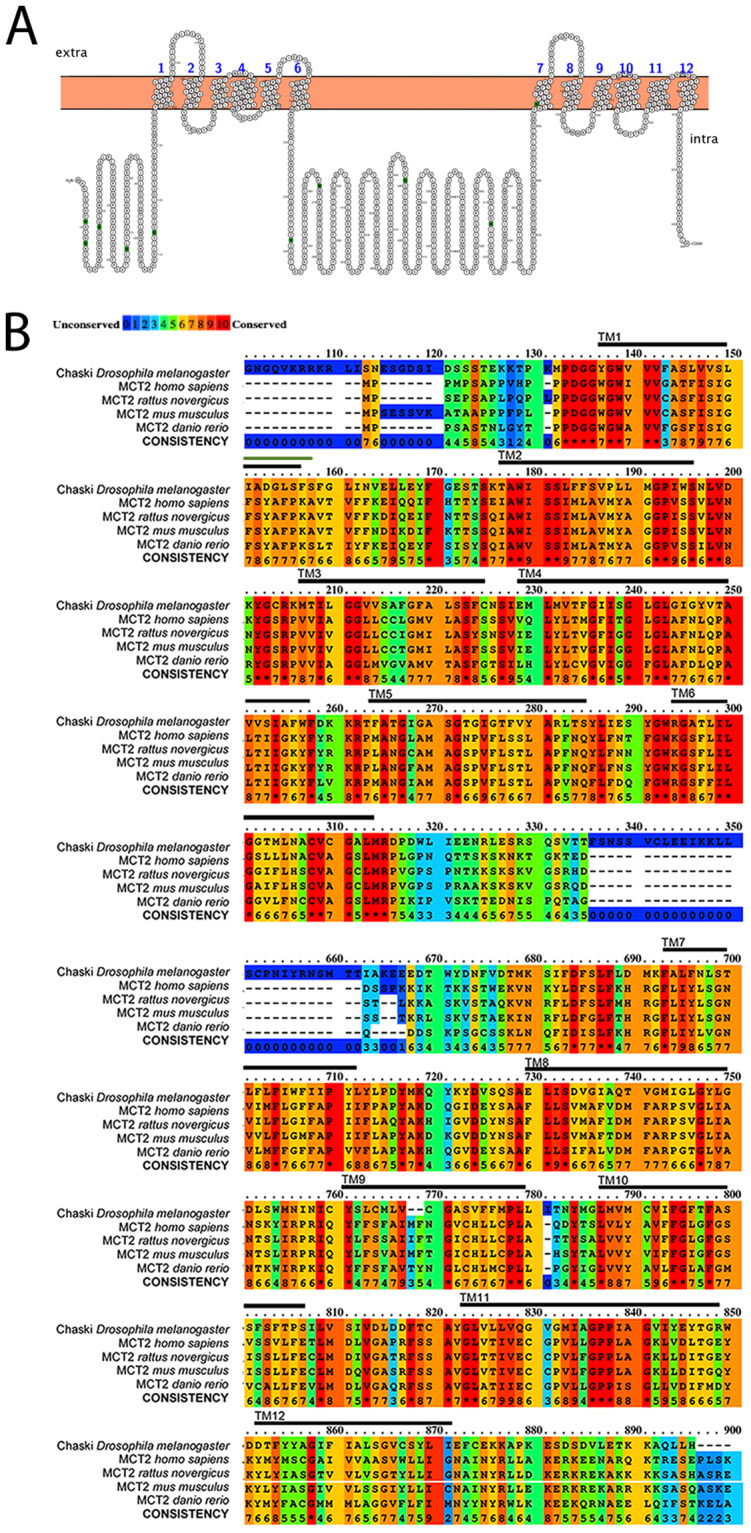
Chaski protein has homology to monocarboxylate transporters. (**A**) Predicted membrane topology with the characteristic 12 transmembrane domains (TM1 to TM12) organized in two groups of six separated by an intracellular loop of the SLC16A family (Monocarboxylate transporters). Green aminoacids represent glycosylation sites (**B**). Comparison of Chaski (first line) with MCT2 from human, rat, mouse and zebrafish. A black line in top of the alignments labels TM domains. The two characteristic MFS (Major Facilitator Superfamily) domains are: MFS1 from aminoacid 135 to 315 overlapping with TM1 to 6 and MFS2 from aminoacid 685 to 869 overlapping with TM7 to TM12. The color code is depicted in the top, from red denoting the conserved aminoacids to blue representing the non-conserved aminoacids.

Chk contains the signature of the Major Facilitator Superfamily 1 (MFS1), domains that characterize membrane transporter proteins^13^ and shows homology to mammalian monocarboxylate transporters (MCTs). Chk hidropathy plot (ProtterServer webservice) predicts 12 transmembrane α-helical (TM) domains with N- and C-termini facing the cytosol and with a large intracellular loop between TM 6 and 7 similar to mammalian MCTs (Fig. 1A). An alignment between Chk, and MCT2 proteins from human and other vertebrates (Fig. 1B) shows that most of the conserved residues are located at the transmembrana domains (TM) of the protein (Fig. 1B indicated with lines on top of the color-alignment), according with what was also described in mammalian MCT family^14^. In contrast, a lower homology is observed in loop regions and hydrophilic regions of the sequences (N-terminus, the loop between TM6 and 7, and C-terminus). The loop between TM6 and 7 has a highly variable size in this protein family and was not included in this alignment.

### *chaski* encodes a lactate/pyruvate transporter

**Figure 2:**
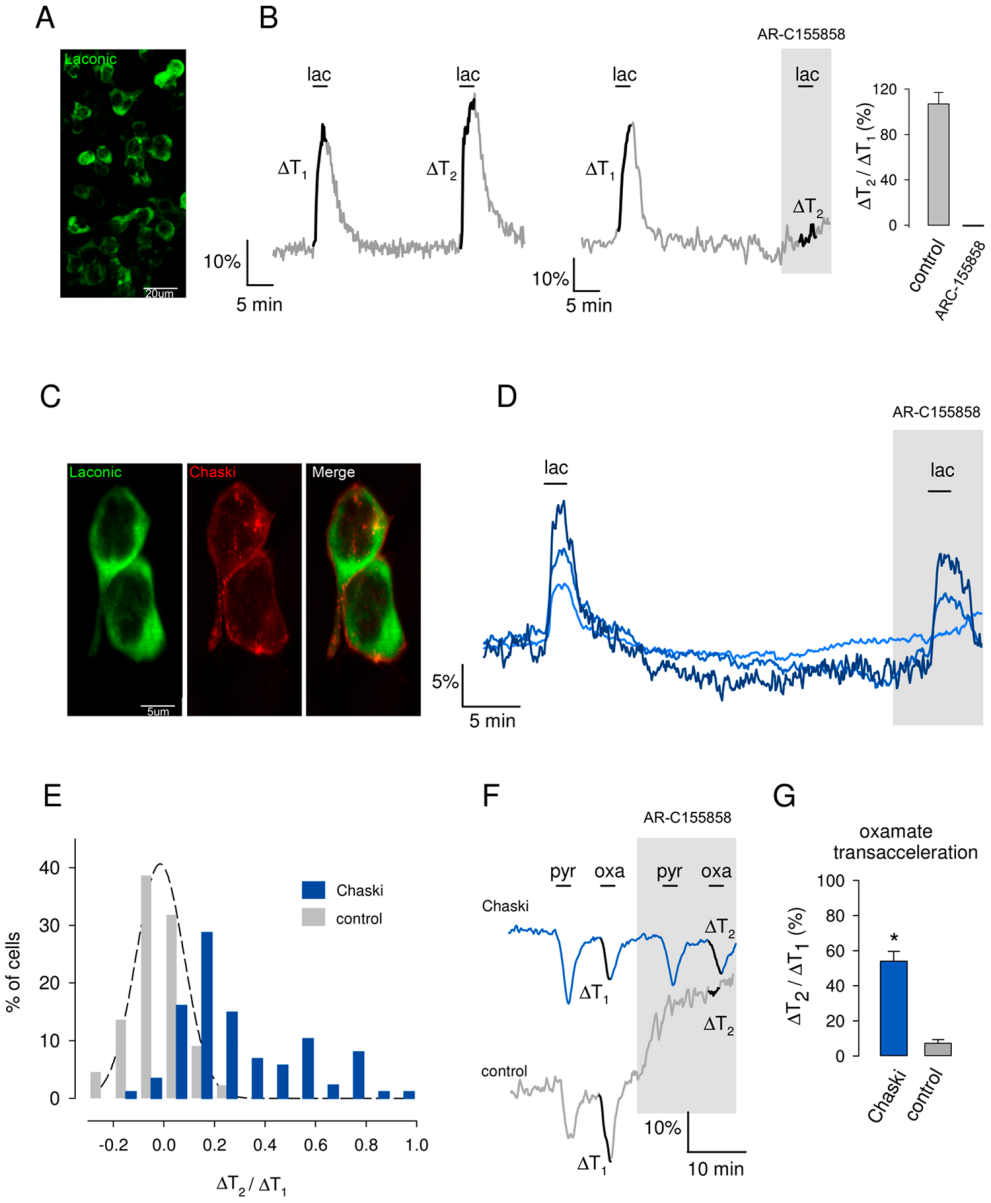
Chaski behaves as a transporter of lactate and pyruvate. (**A**) HEK293 cells expressing Laconic of cytosolic distribution. (**B**) Single cell FRET ratio traces of HEK cells that were deprived of glucose for 30 min and then exposed for 2 min to 10 mM lactate (lac). ∆T_1_ (DT_1_) is the amplitude of the change (%) in Laconic ratio recorded during the first lactate pulse. After lactate washout, the procedure was repeated (DT_2_) in the absence (left panel) or presence (right panel) of the MCT1/2 blocker AR-C155858 (1 μM). Trace segments used to calculate DTs are in black. The bar graph summarizes ΔT_2_/ΔT_1_ for control (41 cells in 3 exps.) and in the presence of 1 μM AR-C15585 (44 cells in 3 exps.). (**C**) HEK293 expressing Laconic and V5-tagged Chk, note the cytosolic localization of Laconic and plasma membrane distribution of Chk. (**D**) Representative single cell traces from a HEK culture co-expressing Laconic and Chk exposed to 10 mM lactate in the absence and presence of the MCT1/2 blocker AR-C155858 (1 μM). (**E**) Frequency distributions of ΔT_2_/ΔT_1_ in control cells (44 in 3 exps.) and *chk*-transfected cells (87 in 3 exps.), estimated from experiments similar to that illustrated in D. The best fit of the Gaussian function y = *a* exp [−(X − *X*_*0*_]^2^/2*b*^2^] to the control data is represented by the interrupted line, with parameters *a* = 41 ± 1, *b* = 0.1 ± 0.003 and *X*_*0*_ = −0.02 ± 0.003, r^2^ = 0.99. (**F**) *chk*-transfected and control cultures were exposed for 2 min to 10 mM pyruvate (pyr) or 6 mM oxamate (oxa) in the absence or presence of 1 μM AR-C155858, data are from representative cells. (**G**) Summary of the responses to oxamate, for control (120 cells in 3 exps.) and *chk*-transfected cultures (139 cells in 3 exps.). Error bars represent s.e.m.; statistic test is a non-paired student t-test. *p < 0.05.

To determine if *chk* functions as a MCT we transfected *chk* cDNA in HEK293 cells and used the lactate-specific FRET nanosensor Laconic to measured lactate flux and infer the transport of lactate and pyruvate through the plasma membrane^15^ (Fig. 2A). HEK293 cells express MCT 1 and 2, both transporters of lactate; this endogenous monocarboxylate transport capacity was blocked with the specific MCT1/2 inhibitor, AR-C155858^16^, which in this cell type effectively inhibits the uptake of lactate and pyruvate^15,17^. Thus, control cells incubated in low extracellular lactate concentration (with low intracellular lactate) displayed a fast increase in the Laconic FRET signal when exposed to increases in extracellular lactate concentration. The same cells showed a slow and sustained increased in the Laconic signal when exposed to the inhibitor; this can be explained by the accumulation of lactate that is continuously produced by the cell, due to the blockage of the endogenous transporters^15^. Additionally, in the presence of the inhibitor the cells do not import lactate from the extracellular media (Fig. 2B).

The Chk protein expressed in HEK293 cells shows a peripheral distribution, consistent with plasma membrane localization (Fig. 2C). Based on Chk sequence divergence with mammalian MCTs we predicted that it would be insensitive to the AR-C155858 inhibitor (for a more detailed explanation see supplemental material). Most of the cells of *chk*-transfected HEK293 cultures exposed to extracellular lactate responded with an acute increase in intracellular lactate (Fig. 2D,E). More important, in the presence of the MCT1/2 inhibitor, there was absence of a slow increase of FRET signal, which is consistent with the view that Chk is insensitive to this reagent and precludes the accumulation of endogenously generated lactate. Notably, the cells with the strongest response in the presence of the blocker also displayed a higher lactate uptake in the absence of the blocker (Fig. 2D), condition in which the artificially expressed protein adds to the endogenous transport function. The histogram in Fig. 2E shows that, in the presence of the blocker, the majority of the cells transfected with *chk* transported lactate at a higher rate than the rate recorded for the control group. In summary, the most parsimonious interpretation of these data is that Chk functions as a lactate transporter and that is insensitive to the MCT1/2 blocker AR-C155858.

To further characterize Chk, HEK293 cells were incubated with glucose to increase intracellular lactate and then the cells were exposed to the monocarboxylate transporter substrates pyruvate (metabolizable) or oxamate (non-metabolizable) to evaluate Chk ability to undergo transacceleration. Transacceleration (or accelerated exchange) is a characteristic of monocarboxylate transporters by which the transport in the manner of a symporter type carrier can be activated by the occupancy in trans, which in this experiment is observed as extrusion of lactate by the import of pyruvate or oxamate (occupancy in trans). As observed in the control trace of Fig. 2F, the addition of pyruvate or oxamate resulted in a rapid and transient decrease of intracellular lactate, as expected of transcacceleration^14,18^. In the presence of AR-C155858 an increase in the cytosolic lactate is observed due to the blockage of lactate extrusion (as in the presence of glucose there is a constant production of lactate that is also constantly extruded through MCTs transporters). While MCT1/2 blockage associated to an effective impairment of transacceleration in control cells, the exposure to AR-C155858 in *chk*-transfected cells only marginally affects it (Fig. 2F,G) further supporting that Chk is a true transporter (as opposed to a channel) able to carry lactate/pyruvate/oxamate transport, sharing its mechanism of transport with mammalian MCTs.

### *chk* is expressed in both neuronal and glia cells

**Figure 3:**
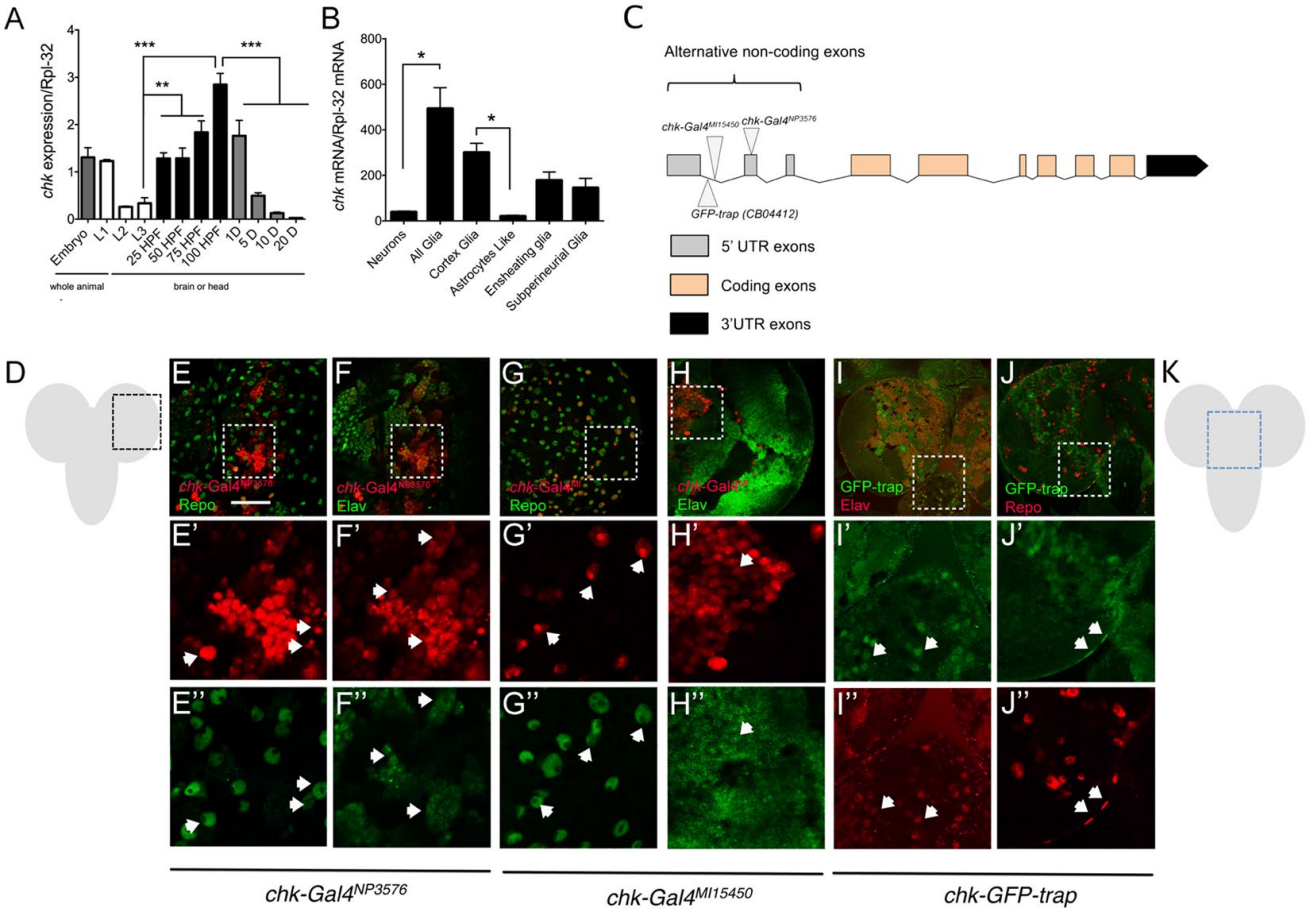
Chaski is expressed in the *Drosophila* central nervous system. (**A**) Determination of *chk* mRNA levels of expression using quantitative RT-PCR from whole embryo and first instar larvae and brain at the indicated developmental stages. *chk* expression reaches a peak at late pupae stage (average of 3 independent experiments from samples in triplicate). (**B**) Evaluation of *chk* expression in different CNS cell types using the TRAP technique. *chk* is found enriched in glia over neurons, and underrepresented in astrocyte-like (Alrm-Gal4) over other glia subtypes, cortex glia (NP2222-Gal4); ensheating glia (NP6520-Gal4) and subperineurial glia (moody-Gal4) (3 independent experiments from samples in triplicate). (**C**) Diagram of the *chk* gene showing the genomic locations of the insertions used as reporters. (**D**) Cartoon of the larval brain boxing the region in the optic lobe where the confocal images were obtained in E to H. (**E–H**)” Analysis of *chk* expression in 3^er^ instar larval brain, using the reporters described in C. (**E,F**)” Representative confocal images of larval brain of animals expressing UAS-NLS-Cherry under the control of *chk*-Gal4^NP3576^ (**E’** and **F’**); co-labeled with anti-Elav (**F**, **F”**) and anti-Repo (E, E”) antibodies to identify expression in neurons and glial cells respectively. (**G,H**”) Representative confocal images of larval brains of animals expressing UAS-NLS-Cherry under the control of *chk*-Gal4^MI15450^ (**G’** and **H’**), co-labeled with anti-Elav (**H”**) and anti-Repo (**G”**) antibodies to identify expression in neurons and glial cells. (**I,J”**). Representative confocal images of larval brains of animals expressing *chk*^*CB04412*^ (GFP-trap, **I’** and **J’**), co-labeled with anti-Elav (**I”**) and anti-Repo (**J”**) antibodies (brain region boxed in **K**). (**K**) Cartoon of the larval brain boxing the region in the central brain from where the pictures in **I** to **J”** were obtained. Expression of *chk* is observed in both REPO and ELAV expressing cells. Error bars represent s.e.m.; statistics applied was Kruskal-Wallis for TRAP analysis and ordinary one-way anova followed by Tukey test for the expression during development. Calibration bar in E is 20 µm. At least four brains for each line were imaged; all displayed the same distribution of immunoreactivity.

Although Chk is known to be expressed in the central nervous system (http://flybase.org/reports/FBgn0033095.html), it is also expressed abundantly in fat body and Malphigian tubule in the adult (flybase.org). To confirm *chk* expression during brain development, we measured expression levels of *chk* using quantitative RT-PCR (qPCR) in different developmental stages. We quantified *chk* expression in whole embryo and first instar larvae and in the brain from two larval stages, three pupal stages and adults. We determined that the higher expression is displayed at late pupae stage (+4 days after pupae formation, Fig. 3A).

To determine which specific cell population in the CNS expresses *chk* we used several different approaches. First, we performed immunoprecipitation of ribosome protein RPL-10 fused to GFP expressed in specific cell types by the GAL4-UAS system followed by the isolation of the associated mRNAs and quantification of *chaski* mRNA by RT-qPCR (TRAP technique^19^). We found that *chk* is expressed in glia cells and in neurons, with a predominant expression in glia. We observed *chk* expression in different glia subtypes^20^, being proportionally more abundant in the cortex glia, which is also known as cell-body-associated glia because it covers neuronal cell bodies. *chk* is also present in astrocyte-like glia, which is rich in neuropiles and surrounds synapses, the ensheating glia that surrounds neuropiles and ensheat and subdivide neuropiles structures and the subperineurial glia, which covers completely the nervous system and is part of the blood brain barrier (Fig. 3B).

We further analyzed *chk* expression in larval brain using different genetic approaches (Fig. 3C and E to J). We evaluated two different Gal4 insertions in the 5′ region of *chk* gene (see a diagram of the insertions in the *chk* gene in Fig. 3C), *chk-Gal4*^*NP3576*^ (inserted in 5′UTR; Fig. E through F”) and *chk-Gal4*^*MI15450*^ (inserted in the first intron; Fig. G to H”) that could represent its endogenous expression (enhancer traps). Additionally we analyzed the expression of *chk* in a GFP-Trap reporter line; *chk*^*CB04412*^ where GFP protein-coding sequence is inserted in the 5′UTR region of *chk* such as the expression of GFP represents the activity of *chk*-promoter (see diagram in Fig. 3C). In all cases we determined that *chk* was expressed in glial and neuronal cells by observing co-distribution of GFP with a pan-glia marker (anti-Repo; Fig. E,G and I) or a pan-neuronal marker (anti- Elav; Fig. F,H and J).

Taken together, these results show that *chk* is expressed in the *Drosophila* brain in glia and neurons and it is comparatively enriched in all types of glia cells.

### *chk* is required for synaptic function *in vivo* and survival to starvation

**Figure 4:**
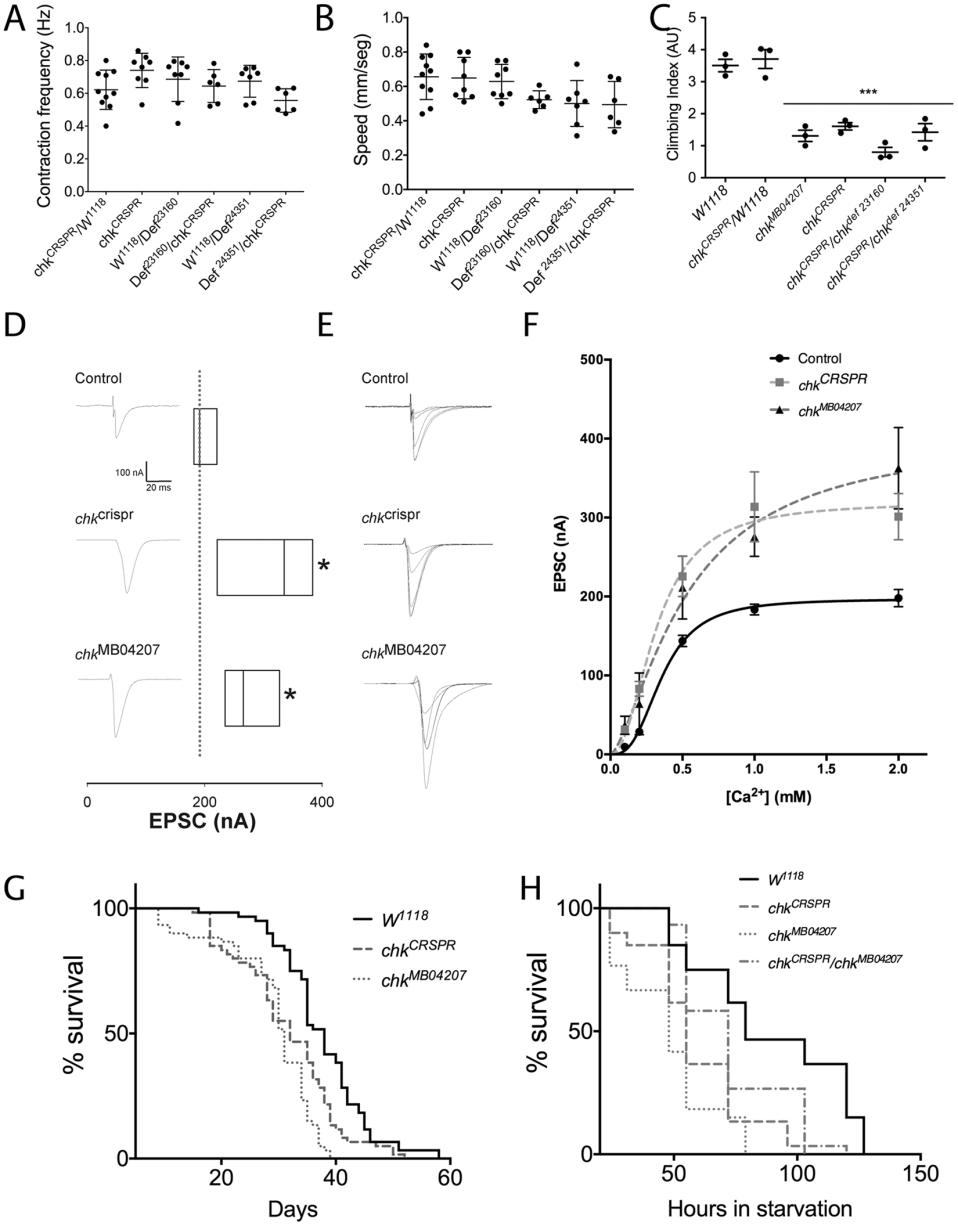
Chaski is required for locomotion in the adult, proper synaptic function and survival under metabolic stress. (**A**) Evaluation of the larval frequency of contraction in *chk* mutants and controls, no differences were found between controls and mutants (10 to 12 larvae per genotype). (**B**) Quantification of the larval speed crawling in different *chk* mutant genotypes, no differences were found between controls and mutants (10 to 12 larvae per genotype). (**C**) Quantification of the climbing index of adult flies 3 to 5 days old of different *chk* mutant genotypes, all *chk* mutants show a decreased climbing compared to controls (3 independent experiments per line, each 20–25 flies of mixed gender; see Fig. S5-C for experiments in males and females separated). (**D**) Representative traces of evoked post-synaptic currents (EPSC) from abdominal muscle 6–7 of 3^rd^ segment exposed to 1 mM external calcium (left) and a plot comparing the average amplitude of the EPSC for mutants and controls. Currents were recorded under two-electrode voltage-clamp with a holding voltage of −80mV, each box represent the mean of 10 stimuli/larvae in 12 larvae/line, *chk*^crspr^ *(p* = *0.027)*, and *chk*^*MB04207*^ *(p* = *0.011)*. Stimuli-artifact was removed. (**E**) Representative traces of EPSC recorded at different extracellular calcium concentrations: 0.1, 0.2, 0.5, 1 and 2 mM calcium from control and mutant larvae. (**F**) Plot of average EPSC amplitudes as a function of external calcium concentration for control and *chk* mutants. Data are shown as mean ± SE, 10 recordings/larvae n = 4 larvae/point. Data from control larvae (black line), and *chk* mutants (grey lines) were fitted to Hill-equation I = I_max_*[Ca^+2^]^n^_H_/((K_0.5_)^n^_H_ + [Ca^+2^]^n^_H_. Differences at 1 and 2 mM concentrations are statistically significant between controls and *chk* mutant flies (two-way anova, p < 0.05 for *chk*^*MB04207*^ and p < 0.001 for *chk*^crspr^). (**G**) Survival curve for control (W^1118^, black continuous line) and *chk* mutant flies (grey broken or dotted lines) obtained in normal food (two independent experiments were pooled, in each 100 flies were reared in vials of 20 flies each of mixed gender). Both mutants show fitted curves significantly different from controls, p < 0.001. (**H**) Survival curves for control (W^1118^, black continuous line) and *chk* mutant flies (grey broken or dotted lines) obtained under starvation (three independent experiments were pooled, in each 60 flies of mixed gender were reared in 3 vials with 20 flies each, see Fig. S5-A and B for experiments in females and males separated). Both mutant curves are significantly different from control, p < 0.001. The transhetorozygous mutant is also different from the control (p < 0.05) Error bars represent s.e.m.; statistic in **C** and **F** is ordinary two-way anova.

To determine the impact of Chk function in the survival and performance of the organism we used two mutant *Drosophila* lines. The first one was an available line, which carries a Minos P element insertion in the coding region of *chk* (*chk*^*MB04207*^, see diagram of the gene in Fig. S1). This insertion associates to a decrease of *chk* mRNA expression to almost undetectable levels, affecting both ORFs (CG45092 and *chk*) predicted in the mRNA (Figs S1 and S2). To obtain a specific decrease of *chk* (without an effect in the second ORF), we used CRISPR technology to generate a small deletion that causes a frame shift and the premature termination of the Chk protein without a destabilization of the mRNA (Figs S2 and S3, *chk*^*CRSPR*^). Both *chk*^*MB04207*^ and *chk*^*CRSPR*^ mutants are viable and fertile. As a general exploration of the nervous system function, we checked the general morphology of the adult brain (3 to 5 - day old flies) and did not find detectable changes (Fig. S4). Then, to test a whole organism function, we tested *chk* contribution to the locomotion performance in larval and adult stages. These tests evaluate the coordination of the movement as well as the strength. In larvae stage both mutants display normal contraction frequency and speed with no clear effect in the coordination (Fig. 4A and B). We evaluated similar parameters in adult flies (3 to 5 days old) in a climbing assay that exploits the natural negative geotaxis of the flies. In this test the flies must repetitively climb ten test tubes in certain time, which requires coordination, speed and resistance. In this assay both mutants showed significantly lower efficiency than control flies (Fig. 4C). In these assays we used a mixture of male and females flies. As it has been reported that male and females could show different behavioral phenotypes, we use only females or males to confirm this finding (Fig. S5) and found the same results.

To more directly asses neural function in the *chk* loss of function condition, we examined the synaptic transmission at the larval neuromuscular synapse (NMJ), which is a well studied model for glutamatergic synaptic transmission and which allows to evaluate a high energy demanding process. We recorded the evoked currents of the larval NMJ using two-electrode voltage clamp in the muscle cell of control and mutant larvae. We observed that *chk* mutant animals exhibited larger evoked postsynaptic current (EPSC) than those recorded from WT synapses (EPSC control = −183.5 ± 3.8 nA, *chk*^*MB04207*^* = −275.8 *± *14.1 nA, chk*^*CRSPR*^ = 313.5 ± 25 nA), at physiological external calcium concentration (1 mM, Fig. 4D). We observed a similar difference when high extracellular calcium (2 mM) solutions were used to bath the tissue. Nevertheless, we did not find significant differences in the amplitude of EPSC between WT and mutant genotypes when recorded at low (0.1, 0.2 or 0.5 mM) extracellular calcium concentrations, (Fig. 4E and F) These observations are consistent with a synaptic transmission defect in the two *chk* mutants which could arise from the inability to manage an increased calcium load in the presynaptic compartment.

Considering the function of *chk* as a lactate/pyruvate transporter we evaluated the general effect of loss of *chk* in the metabolism of adult flies by assessing the lifespan of the mutants in normal conditions as well as their resistance to metabolic stress, measured as the survival of adult flies to starvation (Fig. 4G and H). Both mutants show a small but significant shortening of their lifespan in the standard nutritional conditions (Fig. 4G). On the other hand, *chk* mutants exhibit a marked impairment in their resistance to starvation as compared to WT flies (Figs 4H and S5-C, median survival control = 79 hours; *chk*^*MB04207*^ = 48 hours, ♀-*chk*^*MB04207*^ = 51.5 hours, ♂-*chk*^*MB04207*^ = *48; chk*^*CRSPR*^ = 55 hours, ♀- *chk*^*CRSPR*^ = 55 hours, ♂- *chk*^*CRSPR*^ = 48; WT vs. mutants p < 0.0001).

Defects in locomotion and tolerance to metabolic stress are complex to analyze because multiple tissues and processes are in play. Thus, to advance our understanding of defects associated to *chk* loss of function we focused on specific cell types.

**Figure 5:**
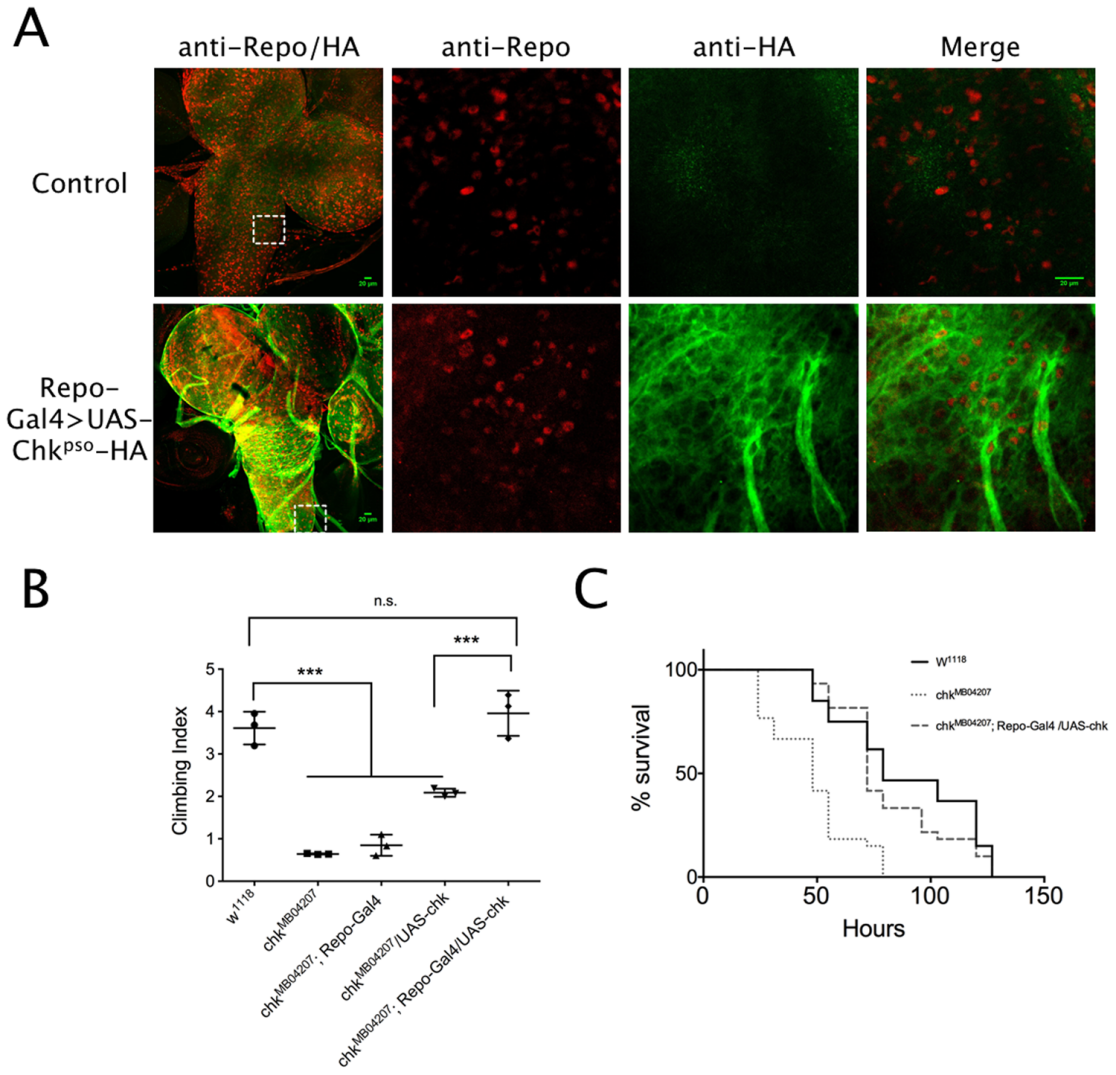
Expression of Chaski in glia rescues mutant climbing defects and sensitivity to starvation. (**A**) Chk subcellular distribution displays membrane localization. First column are representative confocal images of brain and ventral cord from control larva and larva expressing Chk-pseudoscura-HA (Chk^pso^-HA) under the control of Repo-Gal4 immunostained for Repo (red, nuclear localization) and HA epitope (green, membrane localization). Second column, details from the white square in the first image immunostained for Repo (red, nuclear localization), third column same detail immunostained for HA epitope (green, membrane localization), fourth column merge of the two previous images. (**B**) Climbing index for WT, mutant controls and mutants expressing UAS-Chk in glia under the control of Repo-Gal4. The expression of Chk in glia rescues the climbing defects observed in mutants (three independent experiments 20–25 flies of mixed gender each, in duplicate, see rescue experiments in females and males in Fig. 5S-C), (**C**) Survival curves of WT, mutants and mutants expressing UAS-Chk in glia under the control of Repo-Gal4 (two independent experiments were pooled in each 60 flies were reared in vials with 20 flies each). The expression of Chk in glia rescues the decreased survival during starvation observed in *chk* mutants. Error bars represent s.e.m., statistic is one-way anova followed by Tukey post-hoc test. Calibration bar is 20 µm. Experiments were performed without separating males from females.

We first attempted to decreased *chk* mRNA using an available dsRNA (double strand RNA, *chk*^*IR*^) construct (provided by VDRC, also used by^11^). However, by expressing *chk*^*IR*^ in mutant background we concluded that this construct leads to off-targets effects, including lethality when expressed in glial cells, which voids it as a useful tool to pursue these studies. Therefore we used an alternative approach, the selective restitution of Chk protein in mutant backgrounds and focused on glial cell types in which *chk* native expression is most prominent. Thus, to approach the role of Chk in glia cells, we over-expressed Chk in all glia cells using a UAS-Chk under the control of Repo-Gal4 promoter. Additionally we expressed the coding sequence of Chk from *Drosophila pseudoscura* tagged with HA (Chk^pso^-HA) to confirm its *in vivo* subcellular localization. Chk^pso^-HA expressed in glia localizes at plasma membrane, which is consistent with the results obtained with expression in HEK 293 cells (Fig. 5A). In a crucial result, Chk expressed only in glia cells (expression was confirmed by PCR, Fig. S6) was able to completely rescue the climbing phenotype (Figs 5B and S5-C; climbing indexes WT = 3.61 ± 0.223; control mutant Gal4 = 0.849 ± 0.144, control mutant-UAS = 2.09 ± 0.055, rescue in glia cells = 3.96 ± 0.307 mutant vs. rescue p < 0.01) and the starvation sensitivity (Fig. 5C; median survival WT = 79 hours, mutant = 48 hours, rescue in glia cells = 72 hours p < 0.001 mutant vs rescue, WT vs. rescue ns).

These results, together with the observation that Chk is more abundantly expressed in glia cells than in neurons, support a key role of Chk in the metabolism of the cells in the central nervous system of the fly and a fundamental role of glia in the management of metabolites during nutritional stress.

## Discussion

The main finding of this work is the functional identification of a novel transporter for lactate and pyruvate in *Drosophila*, which is expressed preferentially in glial cells over neurons. As expected from its ample expression pattern, loss of *chk* function impacts processes as general, complex and diverse as locomotion in adults and in the survival both in normal conditions and during starvation. In spite of the amplitude of the effect, the sole restoration of Chk expression in glia cells is sufficient to restore those processes, which is consistent with a crucial role of glial transport of metabolites and supports the emerging notion that the brain of insects has evolved a stronger version of ANLS.

The SLC16A family of proteins in mammals is composed by transporters of monocarboxylic acids such as butyrate, lactate and pyruvate, essential products of cellular metabolism. This family has also members that transport other substrates such as thyroid hormones, aromatic aminoacids and carnitin and some synthetic drugs^21^. Chk has been considered, together with dMCT1 and CG11665 to display significant similarities with the most relevant mammalian transporters in the brain^22^. However, the highest homology is observed with Human SLC16A14 (using software: Compara, Isobase and Round-up), which has an unknown function or substrate. Chk additionally displays, as shown in Fig. 1, a considerable homology to SLC7A (MCT2) as well as to MCT1, 3 and 4. Chk, however, shows much lower homology to SLCA2 (MCT8) or SLC10A (TAT), the thyroid hormone transporter and the aromatic amino acid transporter; supporting our results that its substrates are monocarboxylic acids. Our results using heterologous expression of Chk in HEK cells show that it functions as a monocarboxylate transporter that has the capability of translocate lactate, pyruvate and oxamate according to their concentration gradient and the ability to transaccelerate in the presence of substrate in both sides. The molecular mechanism by which this is accomplished was not investigated; it is worth noting that none of the key aminoacids identified for mammalian MCTs1 to 4 for the recognition of the substrate or the H+ linked transport are conserved in the Chk protein^21^ Surprisingly, the phenylalanine identified as the selectivity filter is conserved^21^. Liu *et al*.^23^ proposed that the expansion of vertebrate MCTs from a single gene took place after the divergence with invertebrates, being MCT8 and 10 the most ancient members of the family^23^. The 15 invertebrate MCTs genes, according to this study^23^ would have diverged independently from their vertebrate homologues. Further studies should address if evolutionary divergences have maintained an H^+^ co-transport (like mammalian MCTs) or a novel mechanism of transport is built up in these proteins. Further functional characterization of Chk may inform on the structure and function relationship of the SLC16A family.

Our results show that *chk* is expressed throughout the brain in neurons and glia cells. Additionally the mRNA quantification using the TRAP technique suggests that it is enriched in glia cells over neurons. Although this has to be further studied these results suggest differential distribution of MCTs similar to what is observed in mammals for MCT1 and 2. Two recent reports suggest a major role of MCT transporters in the transfer of metabolites between neuronal and glial cells and a tight coupling between this process and behavior. Thus a decrease in mitochondrial activity favoring aerobic glycolysis in glia associates to increased aggressivity in bees and in flies^24,25^. Moreover flies subjected to a type of training that induces long-term memory showed increased mitochondrial activity in the mushroom bodies (main brain area involved in memory in the fly) without an increase in the glycolytic rate in neurons, suggesting that the mitochondrial substrates are imported from the extracellular^26^. Considering the evidence that glia cells display higher glycolytic activity than neurons and release lactate and alanine^11^, these reports support the key role of metabolite shuttling between glia and neurons where MCT transporters play a major role.

Besides the analysis of expression patterns and functional identification of the Chk protein as a transporter we sought to advance our understanding of the overall role of lactate transport at the organism level, taking advantage of an already available *Drosophila* mutant and a our own construction, which by different molecular mechanism attain a similar loss of *chk* function. Our *in vivo* experiments showed that the two mutants used for functional experiments, *chk*^*MB04207*^ and *chk*^*CRSPR*^, display very similar phenotypes, although *chk*^*MB04207*^ phenotypes appear stronger than the observed in *chk*^*CRSPR*^. It can be inferred from this that the loss of the predicted gene CG45092 function, does not significantly add to the phenotype of the *chk* specific mutant.

*chk* mutant flies display a decreased life span in normal nutritional conditions and under starvation, suggesting that Chk has a unique metabolic function that cannot be compensated by other MCTs expressed in the fly. Most notably, this defect can be rescued by the sole restitution of *chk* expression in glial cells, which is compatible with a major role of glial metabolism in organismal survival, which may be mediated by its ability to provide lactate as an energy substrate to the adjacent neurons.

Consistent with the general view that neuronal function requires the provision of metabolic energy though lactate transport we observed that *chk* mutants exhibit a defect in synaptic transmission measured as increased amplitude of the EJC at physiological calcium concentration. Considering the function of Chk protein as lactate/pyruvate transporter demonstrated here, it is possible to speculate that the synaptic defects are mechanistically related to the metabolic unbalance. This could affect the ability to maintain ionic gradients, one of the most energy demanding processes in the synapse. Plausible explanations for this phenotype include a defect in the extrusion of presynaptic calcium that could lead to higher calcium levels and increased release in response to activation or an increased excitability that results in more calcium entry and the consequent increased release. This is supported by the fact that the defect was evident only at higher extracellular calcium concentration. More studies will be necessary to address the mechanisms that lead to this synaptic phenotype.

The climbing assay provides a more general assessment of the function of the nervous system in the adult *Drosophila*. Using this approach we find a significant impairment of the performance in *chk* mutants, which can be interpreted as a result of nervous dysfunction, although it is not possible to ascertain the level at which it occurs among structures involved in geotactism and locomotion. Nevertheless, and in a strikingly coincident manner with the results discussed above, the reintroduction of Chk protein expression only in glial cells suffices to restore the physiological phenotype, pointing again to a crucial role of glial lactate transport in supporting brain function.

The adult mammalian brain is almost exclusively energized by glucose. However, there is compelling evidence from a diversity of approaches that mammalian neurons not only consume glucose but also lactate, produced by astrocytes from blood-borne glucose^8,10^. In mammals neuronal glycolysis is functionally truncated, so that glucose is preferentially metabolized through the pentose phosphate pathway for antioxidation and protection against apoptosis^27^. In this context, the recent observation in *Drosophila* that genetic truncation of the glycolytic pathway is without apparent functional consequences suggests that insects may have evolved an extreme version of ANLS (astrocyte-to-neuron lactate shuttle). Genetic deletion of MCT1 in oligodendrocytes has been shown to result in white matter degeneration^28,29^, whereas pharmacological and siRNA inhibition of MCTs in the hippocampus resulted in memory deficits in rodents^30,31^. A crucial role for lactate transport and glial MCTs was recently revealed in photoreceptors undergoing degeneration caused by accumulation of lipid droplets induced by mitochondrial failure^32^. Together with the results of Volkenhoff *et al*.^11^, our data speak of a major role for lactate shuttling in *Drosophila* and suggest that division of energy metabolism in brain tissue is a widely conserved evolutionary strategy. It seems therefore plausible that *Drosophila*, with its short life span, rich toolbox of genetic manipulation techniques and relatively inexpensive handling, may prove a useful model for the study of dysfunctional brain energy metabolism, which has been observed at the early stages of neurodegenerative diseases, decades before cell death and the onset of clinical manifestations^33^.

## Materials and Methods

### Bioinformatics Tools

Proposed membrane topology of Chk protein was generated with Protter software (http://wlab.ethz.ch/protter/start/). Multiple sequence alignment between Chk and MCT2 proteins from other species selected from NCBI database was carried out using the PSI-BLAST pre-profile processing (Homology-extended alignment, Praline software, http://www.ibi.vu.nl/programs/praline, IBIVU, Amsterdam). The conservation scoring is performed by software PRALINE where the scoring scheme works from 0 for the least conserved alignment position, up to 10 for the most conserved alignment position. Cladogram was performed using the ClustalW algorithm by using MEGALIGN software (DNAstar Lasergene; Madison, WI).

### Fly strains and constructs

Flies of either sex were raised on standard *Drosophila* media at 25 °C. The following fly strains were used in this study: wild type (w1118), Repo-Gal4, Elav-Gal4, UAS-mCD8-GFP^34^ (Bloomington Stock Center); Rl82-Gal4; UAS-*chk*-RNAi (*chk*^*IR*^, stock #v37141 from Vienna *Drosophila* Research Center, Vienna, Austria), *chk* GFP reporter line (*chk*^*CB04412*^ stock from Fly Trap, currently in Bloomington), glia specific Gal4 lines: NP2222-Gal4; UAS-GFP; moody-Gal4, NP6520 and Alrm-Gal4 were kindly provided by Dr. Mark Freeman (UMASS, Worcester). *chk* null mutant: Minos element insertion *chk*^*MB04207*^ (#24296 stock Bloomington Stock Center), P-element insertion line *chk*^*NP3576*^ (#104545 stock from Kyoto Stock Center). CRISPR mutant was generated following the method of genetically encoding the gRNAs^35^. We designed two gRNAs using the CRISPR optimal target finder tool (http://tools.flycrispr.molbio.wisc.edu/targetFinder/index.php). Both gRNAs sequences are close to the ATG. A BamH1 restriction site near to the target sequences was used later for selection. Sense and antisense oligos with the necessary sequence were acquired from MACROGEN (Seoul, Rep.of Korea), and cloned into the pCFD3 vector using the recommended protocol^35^. This vector has vermillion as selection marker. Flies were generated by Bestgene Inc. (California, USA) using the AttP2 landing site (chromosome 3). For stock generation these flies were crossed to y1v1;;Dr/TM6b,sb (BL 32261). For generation of Cas9 mediated DNA breaks, virgins Nos-Cas9 ZH-2A (on X chromosome, BL 54591) were crossed with males of the gRNA lines (named 1.2 and 2 respectively). 10 males of each F1 (Nos-Cas9; gRNA-1.2/+ and Nos-Cas9;;gRNA-2/+) were crossed individually with IF/CyO virgins (this allowed to remove the Cas9 bearing chromosome). Then, five males from the progenies of each cross were selected and crossed individually with virgins IF/CyO. Individual flies coming from each cross were used for DNA extraction. 1 µl of total DNA was used for PCR (using primers that amplified a 650 bp region bearing the desired mutation). PCR products were then directly digested using BamH1. Lines having clear undigested products (mutants should be heterozygotes) were re-tested using the same protocol. Finally, selected lines were sequenced to confirm the generation of deletions in the Chaski genomic region. The *chk*-Gal4 line was generated using a MIMC insertion in the *chaski* locus according to published protocols^36^. The Gal-4 donor construct was obtained from DGRC (# 1325), and the injection was performed by Bestgene Inc. (California, USA). All flies were stored and crossed at 25  °C.

### Immunolabeling, antibody source, and concentration

Third instar *Drosophila* larvae were dissected in calcium-free saline^37^ and fixed for 10 min with 4% paraformaldehyde, larval brains were stained with primary antibodies: mouse or rabbit anti-GFP, 1:200 (Invitrogen); mouse, anti-Repo 1:50, mouse or rat anti-Elav 1:20 (Developmental Studies Hybridoma Bank), rabbit anti-HA 1:500 (Cell Signaling) followed by fluorescent-coupled secondary antibodies FITC, Alexa 594 or Cy5 1:200 (Jackson ImmunoResearch, West Grove, PA).

After immunocytochemical procedures samples were mounted in Vectashield mounting medium (Vector Laboratories, Burlingame, CA). Images were captured using a confocal microscope (Olympus FV1000) and processed using ImageJ (U. S. National Institutes of Health).

### Molecular Biology procedures

To obtain *chk-*cDNA tagged with V5 in a vector suitable for cell expression, *chaski* gene was cloned using Gateway Clonase (Invitrogen) protocol. Briefly *chaski*-coding sequence was amplified by PCR from the available cloned CDS, LD30953 (DGRC Gold Collections) using specific primers (F-GTGTAAAAGTTGAGCTTTTTTAGTCTCTA; R-CTAGTGTAAAAGTTGAGCCTTCTTAGTCT) and cloned in TOPO-pEntry (Invitrogen). The coding sequence was then recombined with clonase enzyme mix (Gateway Technologies, Invitrogen) according to manufacturers specifications in the vector pCDNA 6.2/V5-DEST Gateway (Invitrogen). The positive clones were then sequenced using specific primers (TACCGGTTCGATCGCTGCCTAGGTTTGCG; CGTAGCCCATACACTGGCGCCACCACA; ATTAGGGTATCATCTGGCGAGCCTATAAC; ATCGAGTTGCGGTGAATGCGCATATCCTT; GTGTAAAAGTTGAGCTTTTTTAGTCTCTA) to confirm the whole sequence.

### Intracellular Lactate Measurements

For cell culture, HEK293 cells were acquired from the American Type Culture Collection and cultured at 37 °C in 95% air/5% CO2 in DMEM/F12 10% fetal bovine serum. Cells were transfected at 60% confluence using Effectine Reagent (Qiagen), with approx. 20% of efficiency. AR-C155858 (Tocri Bioscience, Bristol, UK) was added at 1 mM concentration to the cell medium. The culture cells were imaged with an upright Olympus FV1000 confocal microscope and a 440 nm solid-state laser as detailed previously^38^. Masked ratio images were generated from background-subtracted images using ImageJ software. Lactate cytosolic fluxes in cultured cells were measured using the Fret-based lactate-sensor Laconic as previously described^15^.

Data Analysis and Statistics, Cell domains were outlined using ImageJ (1.46r; NIH). The mTFP channel (with bandpass filter 475/64; Semrock) was divided by the Venus channel (with bandpass filter 542/50; Semrock), and the ratio was normalized to the corresponding baseline. Time acquisition curves are filtered with a moving average of five frames and indicated as mean ± SD. Effects were compared using t tests in R (R Core Team, 2014). p values < 0.05 were taken as the significance limit.

### Tissue-specific expression profiling

Chk expression was analyzed by translating ribosome affinity purification (TRAP) method^19^. Transgenic strains expressing RpL10A, under the control of the UAS promoter (GFP::RpL10A fusion protein) were crossed with each specific Gal4-expressing line. Polysome affinity purification was carried out collecting around 1 mL of flies of either sex, frozen in liquid nitrogen, vortexed to separate body from legs and heads and pass through sieves to collect the heads. Heads were lysed with RIPA buffer (1×) and used 5000 μg of protein per each immunoprecipitation; inputs were 10% of the total. The head homogenize was mixed with anti-GFP conjugated beads (Chromoteck; Planegg-Martinsried, Germany) (25 μl of GFP-conjugated beads per reaction) and lysis buffer up to 1 mL total volume; the samples were incubated for 2 hours in a rotator at 4  °C and were washed 4 times with 0.350 M KCl Buffer for 10 min each at 4  °C. The elution was carried out with 20 μL of TRizol Reagent (1×) for 15 min, the samples were subjected to Quantitative RT-PCR each in triplicate; 0.5 to 1 µg of total RNA was used to generate cDNA using M-MLV reverse Transcriptase and Oligo-dT primer (Promega). Primers and probes were designed for *chk* (F-ATGATACCCTAATGGGCGGAATAGCTC and R-GAGCTTGACAGCATCTGCGAGGTTA) and RPL-32 (F-AGCGCACCAAGCACTTCATA and R-GTGCGCTTGTTCGATCCGTAA) from integrated DNA Technologies. These primer/probe sets were used to quantify expression. Results were obtained and quantified using Mx3000p Qpcr (Stratgene) and Mx Pro QPCR Software. Data from Excel were exported to GraphPad Prism 6 (GraphPad Software, Inc., La Jolla, CA) for statistical analysis.

### Behavioral assays and survival experiments

Most experiments were performed with animals of mixed gender. We measured total distance travelled or body wall contractions, according to published protocol^39^. The locomotion assays were carried out with early 3^erd^ instar larvae, from which 10–12 individuals were selected from each line investigated and placed on a 10 cm plate covered 1% agar with a grid base in the absence of food and with direct light, the latter to induce its displacement. The motion patterns were recorded with a Canon EOS 550D camera under a dissecting scope. From the videos the speed of movement and the frequency of contraction were analyzed. All experiments were performed at controlled temperature and humidity of 22 °C and 45–50%, respectively, the larvae were grown at 25 °C. Climbing assays were performed using 3–5 days old adults in batches of 20–25 flies. The ability to climb against gravity was measured by a countercurrent distribution^40^ and scored as described^41^ using a mixed of males and females flies (same number of each). Climbing experiments were repeated using females and males separated (Fig. S5-C). We did not find significant differences in the performance between males and females.

Survival experiments were conducted in vials containing equal number of males and females flies in triplicate with 20 flies per vial (total 60). The flies were transfer every two days to a new vial and the number of dead flies recorded. Starvation was obtained by transferring 5 days old flies into a vial with agarose 1%, dead flies were recorded every 4 and 8 hours. Two independent experiments were pooled for the analysis. Starvation experiments with mutant flies were repeated with males and females separated. We did not find significant differences between males and females and the results observed using mixed genders. Lifespan was determined using 100 flies (5 vials) using the same procedure but scoring once a day. Two independent experiments were pooled for the analysis.

Statistical significance was obtained using GraphPad Prism 6 (GraphPad Software, California, USA).

### Electrophysiology

Evoked postsynaptic currents (EPSC) were recorded from ventral longitudinal muscle 6 or 7 of A2 or A3 segments of third instars *Drosophila* larvae, under voltage-clamp condition. The *Drosophila larval* body wall preparation was obtained as described^37^ in HL3 solution without calcium added, but maintaining the ventral ganglia. The wall ventral muscles were voltage-clamped at Vholding of −80 mV, using a two-electrode voltage clamp amplifier, (Oocyte-clamp 725, Warner Instruments), using low resistance borosilicate glass electrodes (<10 Mohm), filled with 3 M KCl solution. The glass electrodes were pulled using a P-1000 horizontal puller (Sutter Inst. Co, California, USA). The currents were recorded in indicated Ca2+ concentration, buffer made of (in mM): 128 NaCl, 2 KCl, 4 MgCl2, 5 HEPES, and 36 sucrose, pH 7.0^37^. Data acquisition and analysis were performed through Digidata 1440 drive by pClamp10 software, (Molecular Devices, Inc). Evoked postsynaptic currents (EPSC) were collected by template search detection of current deflection. All saline solution reagents were from Merck.

The fitting of the calcium curves was performed using SigmaPlot Software (Systat Sotware Inc, San Jose, California, USA). Statistical significance was determined using ANOVA followed by Tukey test.

## Electronic supplementary material

Supplementary information
