# Supplementary material for: Chaski, a novel Drosophila lactate/pyruvate transporter required in glia cells for survival under nutritional stress
Source: Sci Rep. 2018 Jan 19;8:1186. doi: 10.1038/s41598-018-19595-5 (PMC5775259; doi:10.1038/s41598-018-19595-5)

Chaski, a novel *Drosophila* lactate/pyruvate transporter required in glia cells for survival under nutritional stress.

Supplementary figures

María Graciela Delgado[1,2], Carlos Oliva[3], Estefanía López[2,4], Andrés Ibacache[2], Alex Galaz[6], Ricardo Delgado[5], L. Felipe Barros[6] and Jimena Sierralta[1,2,4*].

Affiliations:

[1]Department of Neuroscience, Faculty of Medicine, Universidad de Chile, Santiago, Chile.

[2]Biomedical Neuroscience Institute, Faculty of Medicine, Universidad de Chile, Santiago, Chile.

[3]Department of Cell and Molecular Biology, Faculty of Biological Sciences, Pontificia Universidad Católica de Chile, Santiago

[4]*Drosophila* Ring in Developmental Adaptations to Nutritional Stress (DRIDANS), Universidad de Chile, Santiago, Chile.

[5]Department of Biology, Faculty of Sciences, Universidad de Chile, Santiago, Chile

[6]Centro de Estudios Científicos, Valdivia, Chile.

*to whom correspondence should be addressed

Contact Information:

Jimena Sierralta, Professor,

Department of Neuroscience, Faculty of Medicine, Universidad de Chile, Santiago, Chile

Independencia 1027, Santiago 8380453, Chile.

jimena@neuro.med.uchile.cl

Supplementary Figures

Figure S1: Diagram of the *chk* gene showing the position of the ATG for Chk ORF and for CG45092 ORF. The cartoon also shows the position of the insertion of the Minos element MB04207.

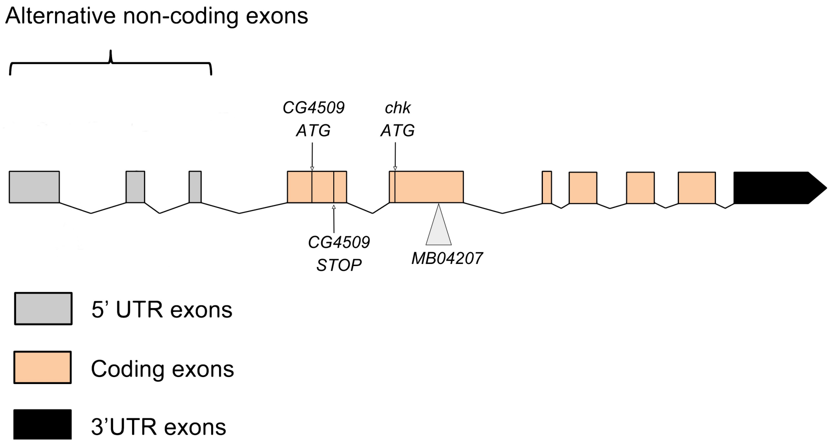

Figure S2: Quantification of *chk* mRNA in mutants and controls by RT-qPCR. Error bars represent SEM, statistic is one-way anova followed by Tukey post-hoc test.

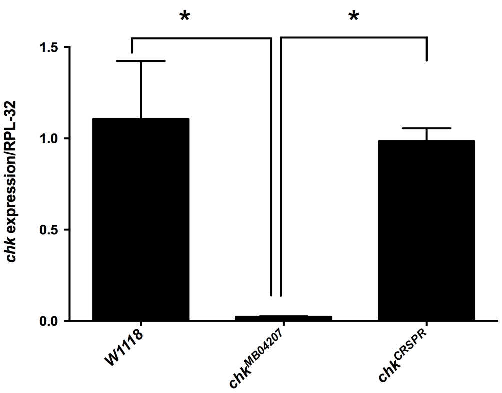

Figure S3: Sequence of the *chk*^{CRSPR} mutant **(B)** and the reference *chk* sequence **(A)** including the translation. In cyan is highlighted the sequence that was deleted and in green the point of religation. The fragmented deleted causes a frame shift generating a truncated protein, see the translation in yellow. **(C)** Alignment of the control and mutant sequence

A: Chaski_REF_ORF_(fragment)

```
atggccaagctagcgacggatccaaacgcctccaacaacaacagctgcgaggcggttaac
 M  A  K  L  A  T  D  P  N  A  S  N  N  N  S  C  E  A  V  N
aacaaccacaatggccagaaccagagccaaaacggaggcgcctccaattaccaggccctg
 N  N  H  N  G  Q  N  Q  S  Q  N  G  G  A  S  N  Y  Q  A  L
cctcttacgcctgctccggccaacacgccactccacaaggccatcaagcacgacctcttc
 P  L  T  P  A  P  A  N  T  P  L  H  K  A  I  K  H  D  L  F
ccggaggtcaccttctgcaacctctccgtagaggagctcgcagatggggctggacacagc
 P  E  V  T  F  C  N  L  S  V  E  E  L  A  D  G  A  G  H  S
cgtgtggtaaggagcagtgtcatcgaactggaggacggcaccatgacgtgcctgatgaac
 R  V  V  R  S  S  V  I  E  L  E  D  G  T  M  T  C  L  M  N
ggaaatgggcaggtgaagcgtcgcaaacgcct
 G  N  G  Q  V  K  R  R  K  R
```

B: Crispr_2_3.4

```
agttgaccaacagagagagggaatatttacctaccgcctacttaagaaaatttatgctgaaa
   L  T  N  R  E  R  E  Y  L  P  T  A  Y  L  R  K  F  M  L  K
tctaatgcaaattgtgttcacttccaataatcacttggacaagaaagtgcaaattttaca
 S  N  A  N  C  V  H  F  Q  -  S  L  G  Q  E  S  A  N  F  T
aacctttttatagacgtagctgaaccctttacacaacaacacgatcactgggactccgac
 N  L  F  I  D  V  A  E  P  F  T  Q  Q  H  D  H  W  D  S  D
atggccaaacgcctccaacaacaacagctgcgaggcggttaacaacaaccacaatggcca
 M  A  K  R  L  Q  Q  Q  Q  L  R  G  G  -  Q  Q  P  Q  W  P
gaaccagagccaaaacggaggcgcctccaattaccaggccctgcctcttacgcctgctcc
 E  P  E  P  K  R  R  R  L  Q  L  P  G  P  A  S  Y  A  C  S
ggccaacacgccactccacaaggccatcaagcacgacctcttcccggaggtcaccttctg
 G  Q  H  A  T  P  Q  G  H  Q  A  R  P  L  P  G  G  H  L  L
caacctctccgtataggagctcgcggatggggctggacacagccgtgtggtaaggagcag
 Q  P  L  R  I  G  A  R  G  W  G  W  T  Q  P  C  G  K  E  Q
tgtcatcgaactggaggacggcaccatgacgtgcctgatgaacggaaatgggcaggtgaa
 C  H  R  T  G  G  R  H  H  D  V  P  D  E  R  K  W  A  G  E
gcgtcncaaacgcctgatctcgaacgagtccggcgactcgatcgactccnntnccccgga
 A  X  Q  T  P  D  L  E  R  V  R  R  L  D  R  L  X  X  P  G
gaaannngttncacggagaaa
 E  X  X  X  T  E  K
```

C:

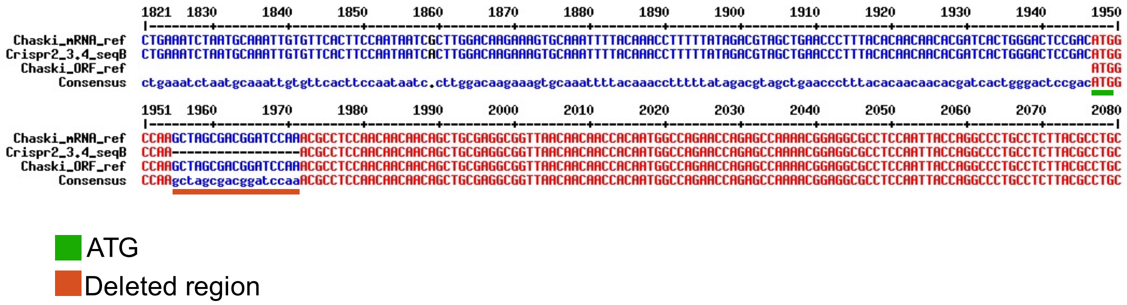
Chaski_mRNA_ref
Crispr2_3.4_seqB
Chaski_ORF_ref
Consensus
ATG
Deleted region

Figure S4

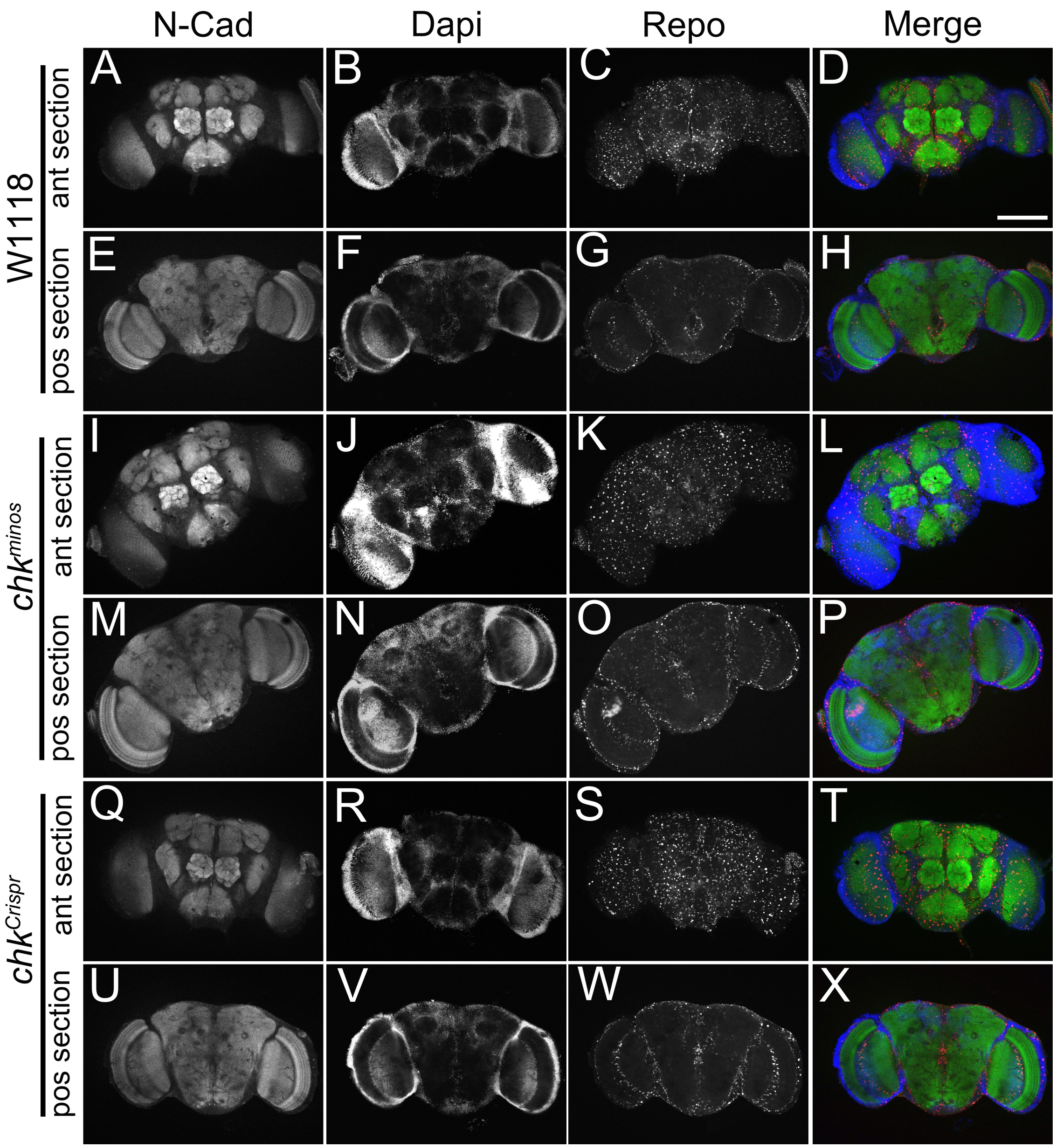

Figure S4: *chk* mutants display normal brain architecture. **(A-X)** *Drosophila* adult brains (3-5 days old) visualized with the indicated antibodies. Single anterior and posterior sections are shown. **(A-H)** W1118 control flies (n = 5). **(I-P)** *chk*^{MB04207} mutants (n = 6). **(Q-X)** *chk*^{CRSPR} mutants (n = 6). Note that general neuropile organization (N-Cadherin), nuclei distribution (Dapi) and glial cells (Repo) are conserved across the different genotypes. Scale bar in D is 150 µm.

Figure S5

Behavioral and survival experiments by gender

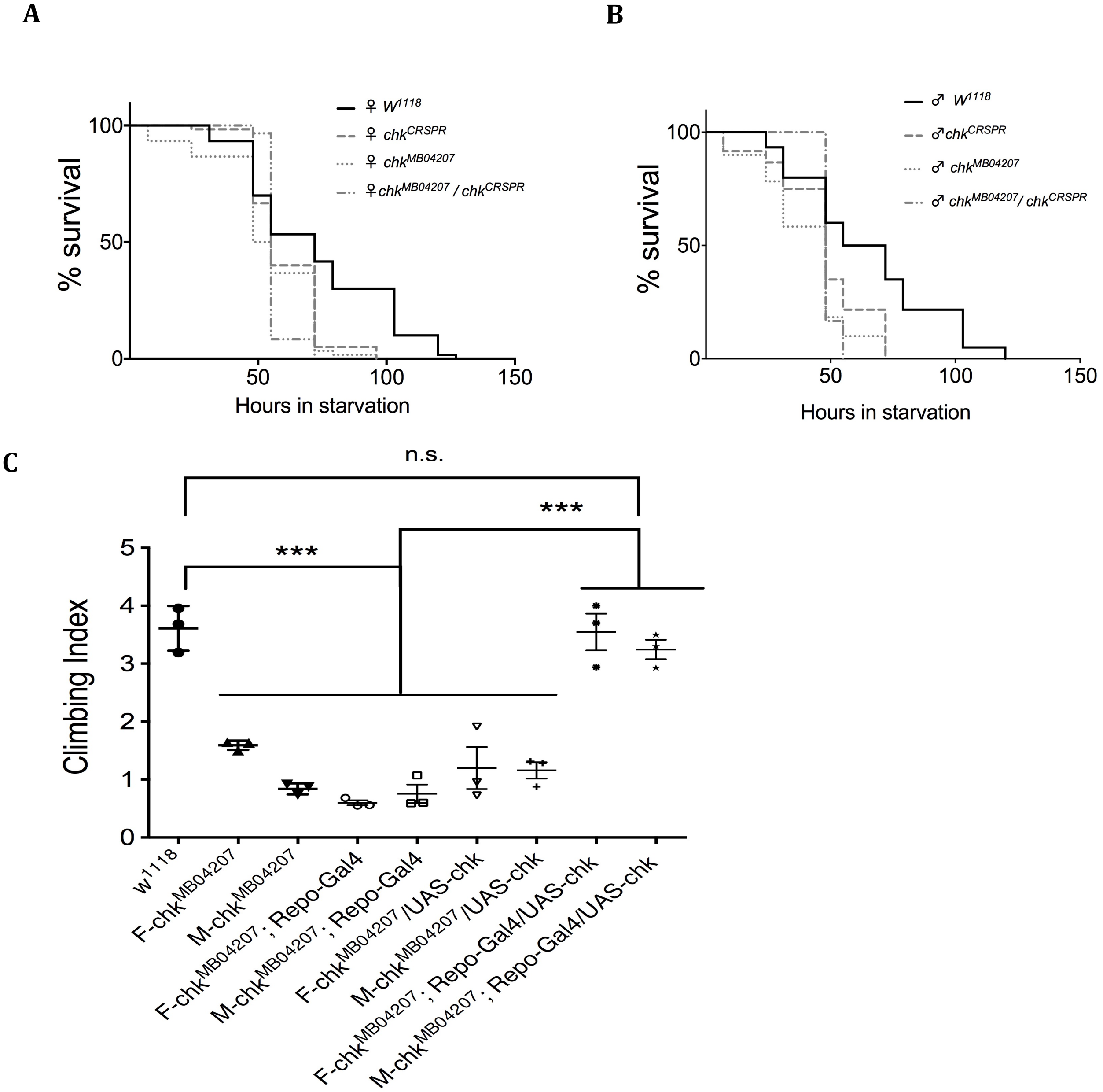

Figure S5:

Behavioral and survival experiments.

Survival to starvation in females (A) or males (B) of different *chk* mutant flies. Flies were separated by sex and then left in vials (25 per vial) with 2 ml of 1% agarose to

avoid desiccation. The number of dead flies was determined every 4 or 8 hours. $p < 0.001$ w^{1118} vs *chk* mutants in males and females.

C. Climbing assay for the different genotypes that represent controls, (mutant + the UAS or GAL4 only) and the rescue experiment using only females or males adult flies. For all the genotypes males and females were not significantly different. Statistic was one way anova followed by tukey, *** $p<0.001$. F: females, M:males

Figure S6

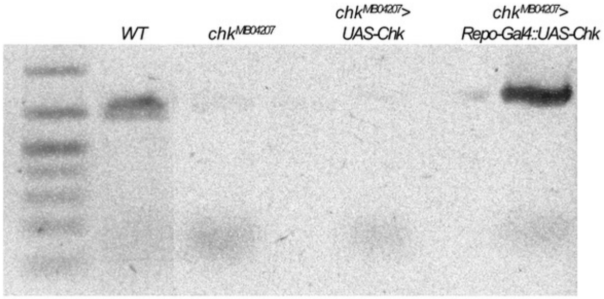

Figure S6: Expression of UAS-Chk mRNA under the control of Repo-Gal4. RT-PCR in control and *chk*^{MB04207} . The PCR was performed with a pair of primers in exon 1 of *chk* gene.

Support for the inability of AR-C155858 to block Chaski.

Nancolas et al., 2015, described the key aminoacidic residues in MCT1 for the recognition by the AR-C155858 blocker. Based on the fact that AR-C155858 blocks MCT1 and 2 but not MCT4, they were able to identify residues that were involved in MCT1 binding to the AR compound. With this strategy they defined that the drug binds to key residues in TM7 to 10 (Ovens et al., 2010) and characterized residues that are important for the direct attachment of the compound as well as residues that interact with the inhibitor in order to allow the access to the binding site located in the cytosolic side of the MCT1/2 protein. The comparison of the sequences between MCT1/2 and Chaski strongly supports the insensitivity of the *Drosophila* protein to the blocker since of the 10 key residues, only one is conserved. From the rest, only three have semiconservative substitutions. Moreover, in MCT4, which is insensitive to the AR-C 155858 blocker, there are five conserved aminoacids, three semi-conservative substitutions and two non-conserved ones. So, Chaski is more divergent from MCT1 even that MCT4, which does not bind the AR compound at all. Below a table modified from Nancolas et al. 2015 and the alignment of MCT1 and Chaski (Fig. S7).

Table 1: Amino acid residues associated with the proposed binding site for AR-C155858 in MCT1 and the comparison to MCT2, MCT4 and Chaski.
MCT1, 2 and 4 are from rat. Modified from Table 2 Nancolas et al., 2015.

Proposed binding site residues			
**MCT1**	**MCT2**	**MCT4**	**Chaski**
Lys^{38}	Lys^{43}	Lys^{40}	$*Phe^{157}$
Asp^{302}	Asp^{297}	Asp^{278}	$**Gln^{739}$
Arg^{306}	Arg^{301}	Arg^{282}	$*Met^{743}$
Phe^{360}	Phe^{255}	Tyr^{336}	Phe^{798}
Ser^{364}	Ser^{259}	$**Gly^{340}$	$*Phe^{802}$
Leu^{274}	Ile^{263}	$*Pro^{250}$	$*Tyr^{711}$
Ser^{278}	$**Ala^{273}$	$*Val^{254}$	$*Pro^{715}$
Met^{65}	Met^{71}	$**Leu^{67}$	$**Phe^{184}$
Met^{69}	Met^{75}	$**Leu^{71}$	$*Pro^{188}$
Asn^{147}	Asn^{153}	Asn^{149}	$**Thr^{266}$

* non conservative substitutions
** semi-conservative substitutions

References:

Nancolas, B., Sessions, R.B. and Halestrap, A.P. (2015) Identification of key binding site residues of MCT1 for AR-C155858 reveals the molecular basis of its isoform selectivity. Biochem. J. 466, 177-188

Ovens, M. J., Davies, A. J., Wilson, M. C., Murray, C. M. and Halestrap, A. P. (2010) AR-C155858 is a potent inhibitor of monocarboxylate transporters MCT1 and MCT2 that binds to an intracellular site involving transmembrane helices 7–10. Biochem. J. 425, 523–530.

Figure S7: Alignment of MCT1 and Chaski protein. Boxed are the aminoacids identified in MCT1 as important for the action of the MCT1/2 inhibitor AR-C155858.

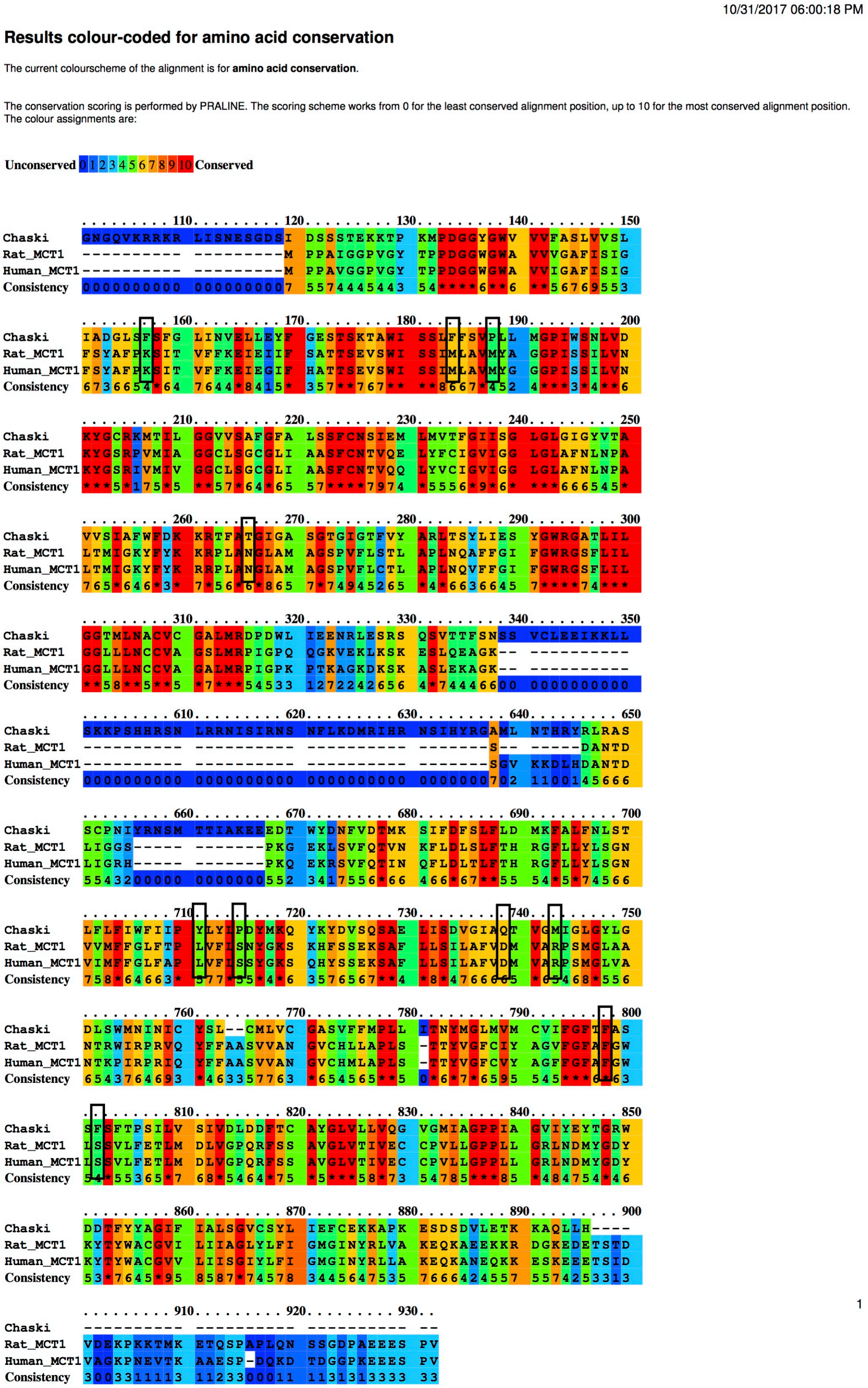

Supplement: Supplementary file 1 — Supplementary information [file 41598_2018_19595_MOESM1_ESM.pdf]
